# Study on the damage characteristics of overburden of mining roof in deeply buried coal seam

**DOI:** 10.1038/s41598-022-15220-8

**Published:** 2022-07-01

**Authors:** Tianwen Long, Enke Hou, Xiaoshen Xie, Zhigang Fan, Ermin Tan

**Affiliations:** 1 College of Geology and Environment, Xi’an University of Science and Technology, Xi’an, 710054 China; 2 Shaanxi Provincial Key Laboratory of Geological Support for Coal Green Exploitation, Xi’an, 710054 China; 3 Shaanxi Binchang Wenjiapo Mining Co., Xian Yang, 713504 China

**Keywords:** Hydrology, Solid Earth sciences

## Abstract

The study of water-conducting fracture zone development height is key to the scientific prevention and control of water damage in mines. Based on the geological conditions of the Wenjiapo coal mine in Binchang, China, this paper investigates the development of water-conducting fracture zone in overlying bedrock during mining under large buried depth and huge thick aquifer by combining on-site well-location microseismic monitoring and laboratory similar material simulation. To overcome the limitation of the " limited outlook " of water-conducting fracture zone investigation, the spatial development characteristics of roof fissures in coal seam mining were determined by on-site " the underground - ground" combined microseismic monitoring and follow-up monitoring, and the development of overlying rock fracture under the large depth of burial was concluded. The fractures were mainly distributed in the upper part of the protective coal pillar on both sides of the working face, but less in the upper part of the working face, and primarily distributed in the protective coal pillar on the side of the working face and the adjacent mining area. To verify the accuracy of the conclusion, the overlying bedrock movement and deformation characteristics and the development process of the hydraulic fracture zone during coal seam mining were analyzed by simulating similar materials in the laboratory, using the monitored area as a prototype. The results show that the development height of the mining fracture zone obtained from microseismic monitoring is basically consistent with the simulation results of similar materials. The research finding have significant implications for the study of fracture distribution characteristics and the evolution law of mining overburden, and provide a foundation for scientific prevention and control of water damage on the roof.

## Introduction

In the process of underground coal resource recovery, the retrieval of the working face disrupts the original stress balance of the surrounding rock, and the redistribution of the rock stress causes deformation and destruction of the surrounding rock^1–3^. Before destroying the rock body, energy is inevitably released, resulting in acoustic emissions and microseismic phenomena^4^. In the process of back mining at the working face, the rock stress around the mining void area and hydraulic pillar is adjusted, and the surrounding rock is deformed and destroyed^5^. During the process of deformation and damage, the microseismic produced by the underground rock body is one of the physical effects of the energy release process of the coal rock body, and the location and intensity of the microseismic occurrence reflect the stress state and damage degree of the overlying roof of the coal seam to a certain extent^6,7^. Therefore, the collected microseismic signals are processed, analyzed and studied in combination with the geological parameters of the roof, which can be used as a decision basis for the development height of the fracture zone of the mining overburden roof^8,9^. Compared with the traditional empirical formula method, piezometric test, borehole peeping and flushing fluid consumption and unilateral downhole microseismic monitoring, the combined "underground-ground" microseismic monitoring technology improves the positioning accuracy of microseismic data in elevation, and this method is regional monitoring, with an extensive monitoring range, flexible measurement points^2,10^. The method is a regional monitoring method, with the characteristics of a monitoring range, flexible deployment of measurement points, real-time dynamic monitoring, and a large amount of information about the spatial distribution characteristics of roof fracture development that can be revealed by the results^11,12^. Compared with surface or roadway microseismic monitoring, "surface-roadway" combined microseismic monitoring is more accurate in spatial positioning and more suitable for the investigation of the water-conduction fracture zone. Compared with the common exploration, "surface-roadway" combined microseismic monitoring can reflect the process of water-conduction fracture zone development in the region, which is more in line with the practical needs of engineering.

In addition to in situ detection, physical similarity simulation on the experimental platform through similar materials with similar ratios is also a vital means, and it is widely used in the study of mine pressure and overburden dynamic changes due to its economy, intuitiveness, and ease of operation^5,13–16^. Some scholars have used the Ashby diagram to analyze the stability of racks during the coal development process. They proposed new rock parameters to provide a new method for evaluating geological materials^17^. A physical model test was carried out with the working face passing through a fault. Then, the effect of mining on fault activation slip was studied, the influencing factors of mining height, water pressure, aquiclude thickness and excavation distance and the response of basal stress and fault plane stress in the coal body during fault slip were analyzed^18,19^. The study revealed that inrush occurred most readily at the open-off cut or mining face.

Because there is no accepted formula for calculating the water-conduction fracture zone in the Binchang mining area, the predicted value of the mine water surge deviates from the actual value. Mining overburden fracture zone development height prediction has become one of the impediments to engineering and scientific research because the Binchang mining area has yet to form a unified recognized formula for calculating the water-conduction fracture zone^20^. After the workface mining, the overlying rock layer in the mining void area produces tension and pressure damage and forms mining fissures and sinks. The release of energy during the destruction of the surrounding rock in the mining fracture zone is the main source of mine pressure^21^. In the twenty-first century, microseismic monitoring technology is a newly emerged physical monitoring technology that has been successfully applied to early warning in several mine disasters, such as impact ground pressure monitoring, mine water damage control, gas extraction, and coal and gas protrusion in China. This technology can provide the spatial location, time, and energy magnitude of rock rupture, allowing for mining overburden damage monitoring and analysis of mining overburden movement characteristics.

To this end, the author carried out a series of studies in theoretical, experimental and water control technologies and conducted a field monitoring study on the fracture development of mining overburden using the YJT3 microseismic monitoring system developed independently by China Coal Science and Industry Group Xi'an Research Institute Co. Based on the on-site microseismic monitoring of the Wenjiapo coal mine in Binchang, the author conducted a comprehensive study on the fissure development characteristics of mining overburden in the study area using similar material simulation experimental data.

## Overview of the study area and its geological conditions

### Description of the study area

**Figure 1:**
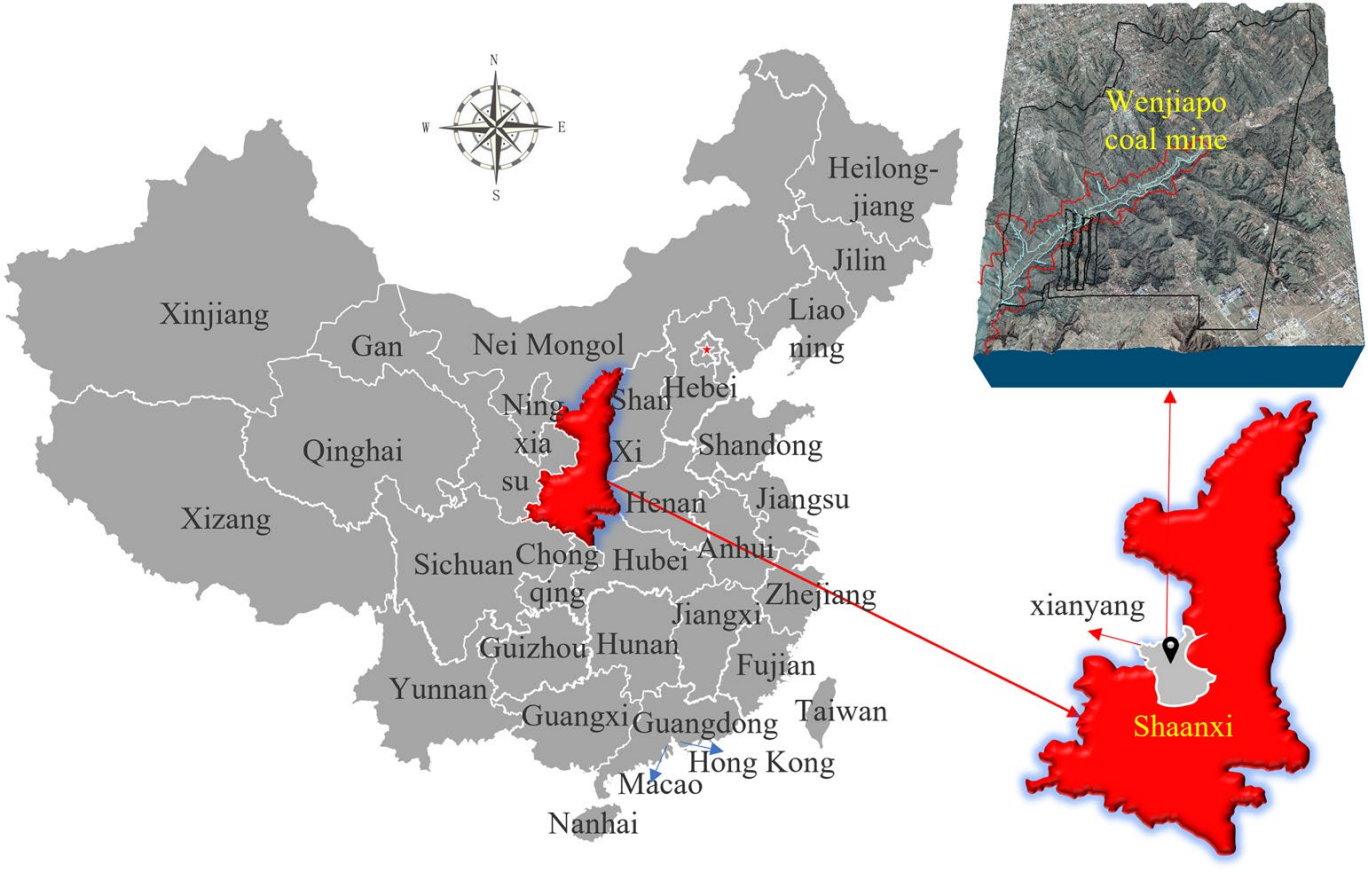
Study area location (China administrative division data from geospatial data cloud: https://www.gscloud.cn/sources/index?pid=3&rootid=3, mapping with ArcGIS 10.2 software).

Wenjiapo coal mine was chosen as the study area, it is located in Shaanxi Province, China, within Latitudes 35°9′18.74″–35°12′8.69″ N, and longitude 108°6′52.40″–108°8′29.42″E (Fig. 1), in a mid-latitude plateau area with a warm temperate semi-arid continental monsoon climate, with four distinct seasons. The study area belongs to Shaanxi Coal Group and is one of the key mining areas in China's 13th Five-Year Plan. The Wenjiapo mine is about 9.86 km wide from east to west and 11.69 km long from north to south, covering an area of 79.6903 km^2^. The elevation of + 1170~+ 1200 m.

### Geological overview of the study area

Wenjiapo coal mine 4103 working face coal seam buried depth 680–690 m average thickness 3.8 m, and average elevation 395 m, dip angle 3°–4°, for nearly horizontal each layer, working face direction length is 2800 m and tendency length 240 m. This monitoring area starts position 282 m from the down-distance cut, transport down-distance cut 290 m, and monitoring push mining distance 520 m. The topography of the monitoring region is typical of a mountainous area. Loess plateau terrain is all covered by the fourth system loess, and the thickness is large, the area is ditch, and beam staggered distribution. The height difference is about 100 m. Due to weathering, denudation and the water scouring effect, the thickness of loess development is extremely uneven, and the terrain gradually rises from north to southeast. In other words, along the direction of back mining in the monitoring region, both the thickness of the loess layer and the depth of the coal seam buried by this influence rise continuously.

**Figure 2:**
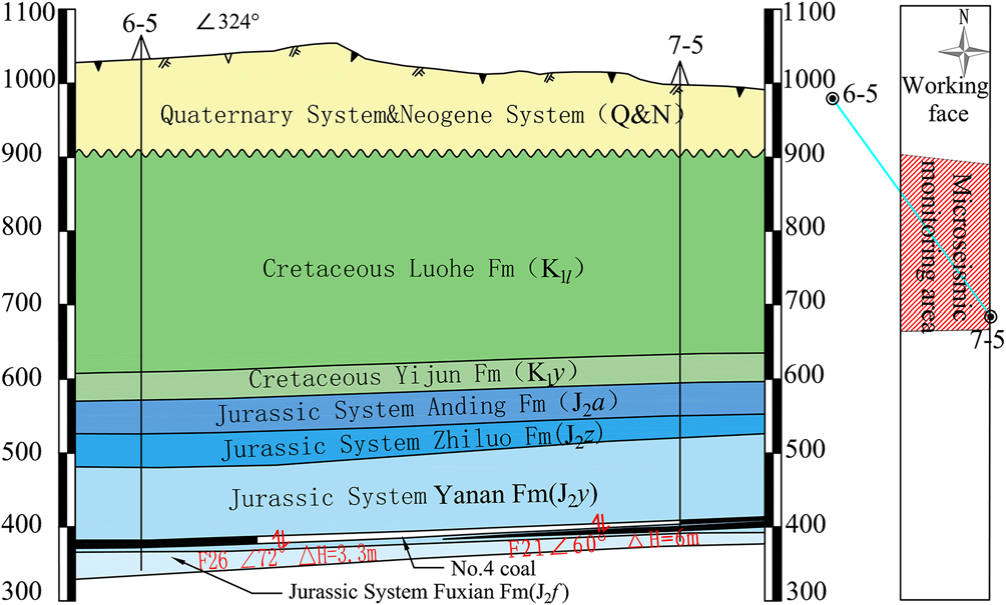
Hydrogeological slope map of monitoring area.

According to the drill holes (6-5, 7-5) near the 4103 working face and the contour map of stratum thickness, the distance between coal and the main water-bearing/water-insulating layer is made diagonally through the direction of the monitoring area (Fig. 2). The average thickness of the aquifer of Luohe Fm is about 275 m, and its bottom is 205–220 m from the top plate of coal 4. From coal 4 upward, there are mainly Yanan Fm aquifer, bottom aquifer of Zhiluo Fm, Yijun Fm aquifer, and Luohe Fm aquifer, and between Yijun Fm and bottom aquifer of Zhiluo Fm There is a mudstone relative water barrier of the Anding Fm with a thickness of about 50 m. The physical and mechanical parameters of the coal strata are shown in Table 1.

**Table 1:** Mechanical parameters of coal measures strata.

Rockiness	Thickness (m)	Modulus of elasticity (MPa)	Compressive strength (MPa)	Poisson's ratio	Self-weight (N/mm^3^)	Friction angle (°)
Loess	100	800	1.67	–	1.21	30
Conglomerate	14	15,000	22.25	0.3	1.32	32
Medium-grained sandstone	86	4130	12.31	0.31	1.04	36
Coarse-grained sandstone	180	5420	18.73	0.32	2.03	32
Conglomerate	36	13,500	16.37	0.29	1.64	38
Sandy mudstone	20	11,021	9.82	0.36	1.37	34
Coarse-grained sandstone	20	7240	8.61	0.39	1.02	30
Mudstone	22	15,362	7.32	0.39	1.18	30
Sandy mudstone	18	11,001	10.63	0.24	1.67	34
Coarse-grained sandstone	12	7240	15.12	0.32	1.60	38
Mudstone	12	13,824	5.32	0.37	1.34	31
Fine-grained sandstone	6	11,123	10.98	0.24	1.86	36
No.4 coal	4	6160	5.11	0.46	1.64	37
Mudstone	20	18,900	4.65	0.39	1.98	36

## Microseismic monitoring of roof slab fracture development

### Microseismic monitoring system arrangement

The microseismic instrumentation adopts the YJT3 microseismic monitoring system independently developed by China Coal Science and Technology Group Xi'an Research Institute Co., Ltd. to monitor the development of the hydraulic conductivity rift zone in the study area of 4103 working face in real time and to locate and analyze the received microseismic events at the same time. This microseismic monitoring system mainly consists of a geophone, a large wire, a collector, microseismic pre-processing software and microseismic event location software. According to the needs, detectors with different sensitivities are selected. Since the coal seam in the monitoring area is buried more profoundly and the thicker Quaternary loess layer is not conducive to the propagation of seismic waves, a geophone with high low frequency and sensitivity of 4.5 V-s/m is selected this time.

**Figure 3:**
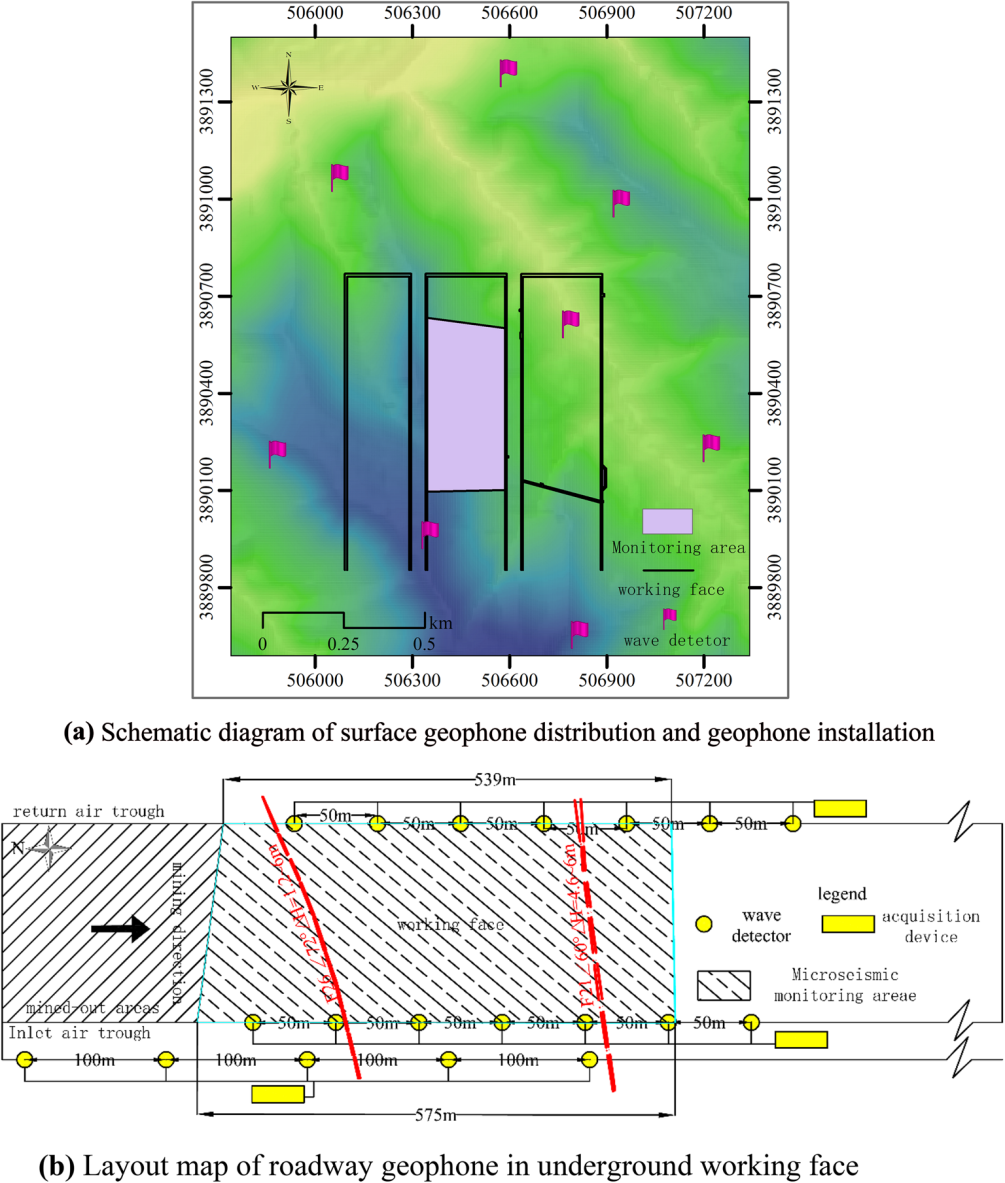
Microseismic monitoring system arrangements.

According to the geological conditions of the monitoring area, the burial depth of the coal seam and the retrieval situation, eight geophones were arranged within about 800 m of the ground in the monitoring area according to the topography and traffic conditions, and the ground was manually excavated with a 0.5 m pit, and the geophone tail vertebrae were replaced by 1.5 m steel bars to improve geophone coupling with the loess layer (Fig. 3a). The coordinates of each measurement point were accurately measured by RTK, a high-precision measuring instrument.

Simultaneously, three geophones are arranged in each of the 4103 working face, transport chute, and 4104 working face return wind chute.The 4103 working face return wind chute and transport chute geophones are 50 m apart, the two lanes are staggered, and the geophone positions are moved according to mining progress. The 4104 working face return wind chute geophones are 100 m apart and are always arranged on the side of 4103 mining area with each lane. The three geophones are connected to the X, Y, and Z axes of a collector with shielded wires, and the coordinates of each measuring point are read using the mining project plan (Fig. 3b).

### Microseismicic positioning principle

The elastic wave signals generated by rock rupture are accepted by multiple geophones, and the time difference and wave velocity are used to localize the microseismic events.

1 $$(x_{i} - x_{0} )^{2} + (y_{i} - y_{o} )^{2} + (z_{i} - z_{0} ) = (T_{i} - T_{0} )^{2}$$

where is *x*_*i*_, *y*_*i*_, *z*_*i*_ the coordinate position of the *i* geophone, *v*_*i*_ is the wave velocity measured by the *i* probe, *T*_*i*_ is the time measured by the *i* geophone, *x*_0_, *y*_0_, *z*_0_ the coordinate of the source and *T*_0_ is the moment of rupture.

There are four unknowns in the equation, and at least four equations are needed, so at least four geophones are needed to locate the microseismic events, and the more geophones there are, the higher the theoretical accuracy of the microseismic event location.

The amplitude of the waveform determines the energy of a microseismic event; the greater the amplitude, the greater the energy, and vice versa.

## Spatial damage characteristics of the roof fracture zone

### Statistical analysis of microseismic event description

**Figure 4:**
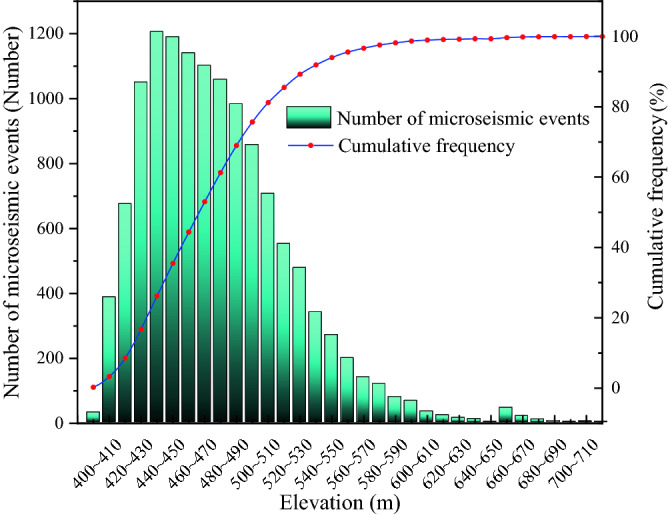
Histogram of quantitative distribution in elevation interval of microseismic events.

Wenjiapo coal mine 4103 working face started pushing on June 4, 2018, and the microseismic monitoring period lasted from July 20, 2018 to October 10, 2018, with a monitoring pushing the length of about 520 m. During the monitoring period, two faults F26 and F21, with a fault distance of approximately 6.8 m, were crossed. After excluding the events with a low signal-to-noise ratio and significant positioning errors, a total of 12,778 microseismic events were located during the monitoring period. The location elevation of microseismic events was divided by every 10 m interval, and the statistical analysis of microseismic events was carried out (Fig. 4).

In Fig. 4, it can be seen that microseismic events occur in the interval of elevation 390–600 m, mainly concentrated in the elevation interval 410–540 m, and microseismic events basically no longer develop upward after elevation greater than 580 m. A very small number of microseismic events occurred after elevation greater than 600 m, which can be neglected. According to the distribution characteristics of the number of microseismic events in different elevation intervals, it can be roughly divided into three ranges: the growth rate is basically the same between elevations of 390–440 m, and the growth rate is basically stable after elevations of 440–500 m and starts to decrease gradually after 500 m; the number of microseismic events at elevations > 550 m is small, accounting for only 5.97% of the total number.

**Figure 5:**
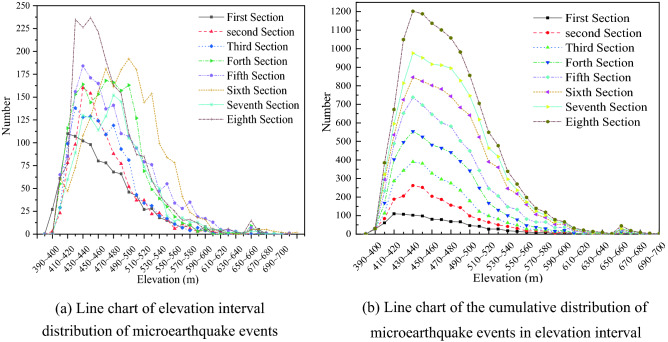
Interval distribution map of the number of microseismic events at an elevation.

From the beginning to the end of the monitoring data, the distribution characteristics of microseismic events were divided into eight segments with a time unit of weeks, and the interval and cumulative number of microseismic events in different height intervals within different segments were plotted (Fig. 5). During the monitoring period, the distribution characteristics of the weekly microseismic events in each growth phase, maximum, stability, and decline were consistent.

From the analysis of Fig. 5, the microseismic events belong to the stage of rapid increase between 400 and 440 m, and the microseismic events reach the peak at elevation 440 m; they basically remain stable during the elevation of 440–500 m, and start to decline sharply between the elevation 500–550 m. According to the growth characteristics of the number of microseismic events at the later stage of monitoring, the number of microseismic events stopped increasing after 520 m. Therefore, the top plate elevation stabilized state at about 520 m, and the rupture signal tended to stop developing upward.

**Figure 6:**
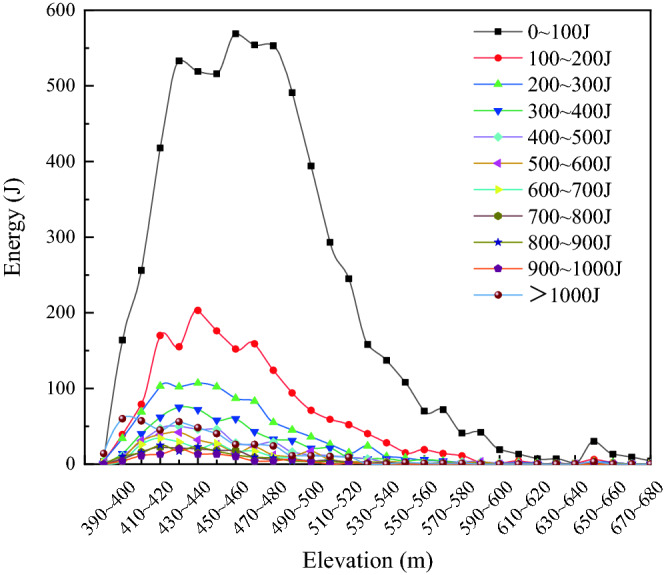
Energy distribution of microseismic events.

The energy level of microseismic events is Joule/J, and an energy size varies from 0 to 3800 J. The energy of microseismic events is counted at intervals of 100 J, and the distribution of the number of microseismic events in different height intervals is shown in Fig. 6, where there are 6249 microseismic events with most of the event energy in the range of 0–100 J, which are small energy events and are distributed in the range of 380 m to 670 m in elevation. The number of microseismic events larger than 1000 J was 445, accounting for about 0.39% of the total number of microseismic events. The number of large energy events accounted for a small percentage and was mainly concentrated between 390 and 470 m, with the maximum value occurring between 390 and 460 m.

### Analysis of spatial distribution characteristics of microseismic events

The spatial and temporal distribution characteristics of microseismic events can reflect the location and sequence of rock rupture, and the density of microseismic events (the number of microseismic events projected on a certain plane per unit area.) can indicate the degree of fracture aggregation of rock fragmentation, according to which the process of rock rupture and fracture zone formation can be analyzed.

**Figure 7:**
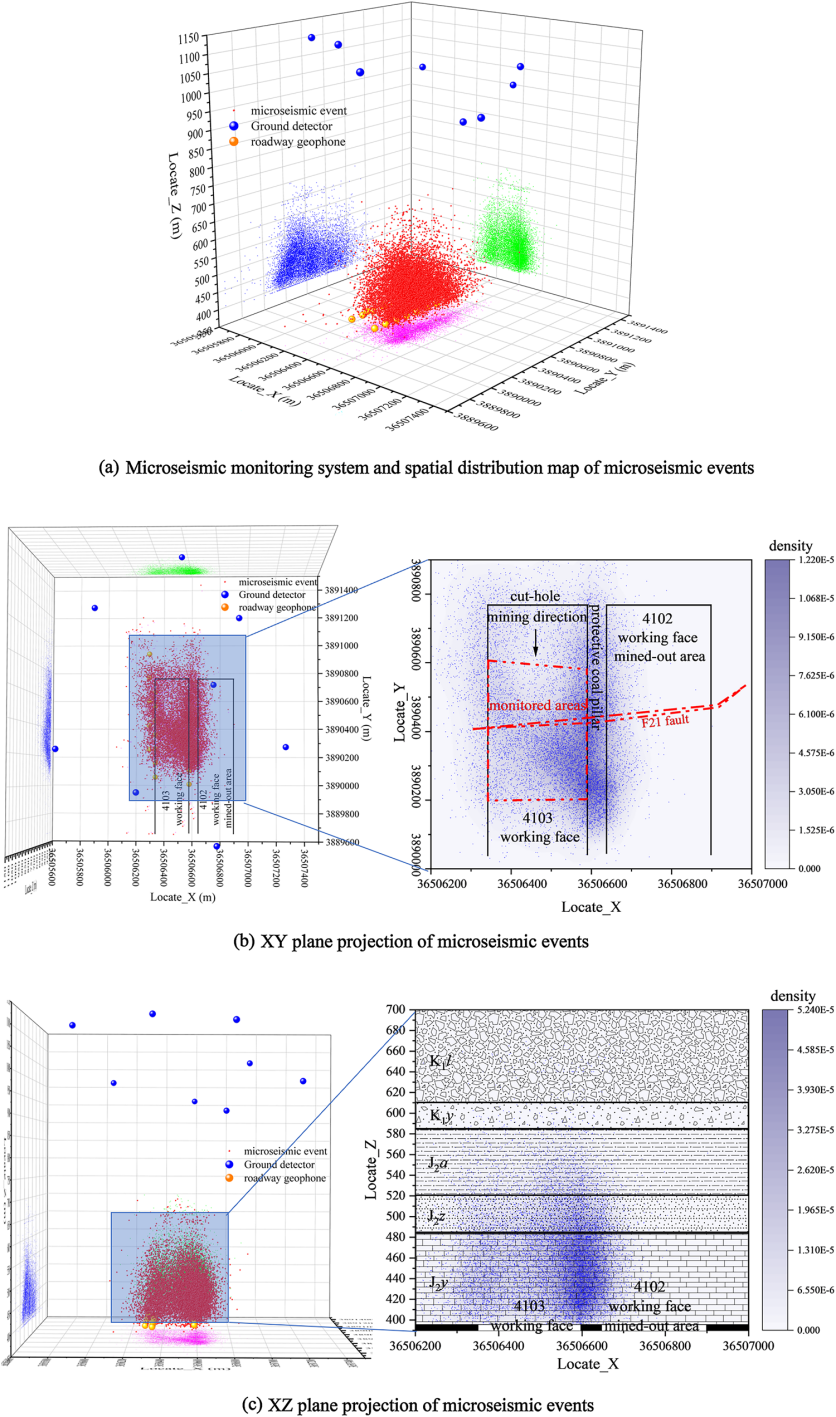

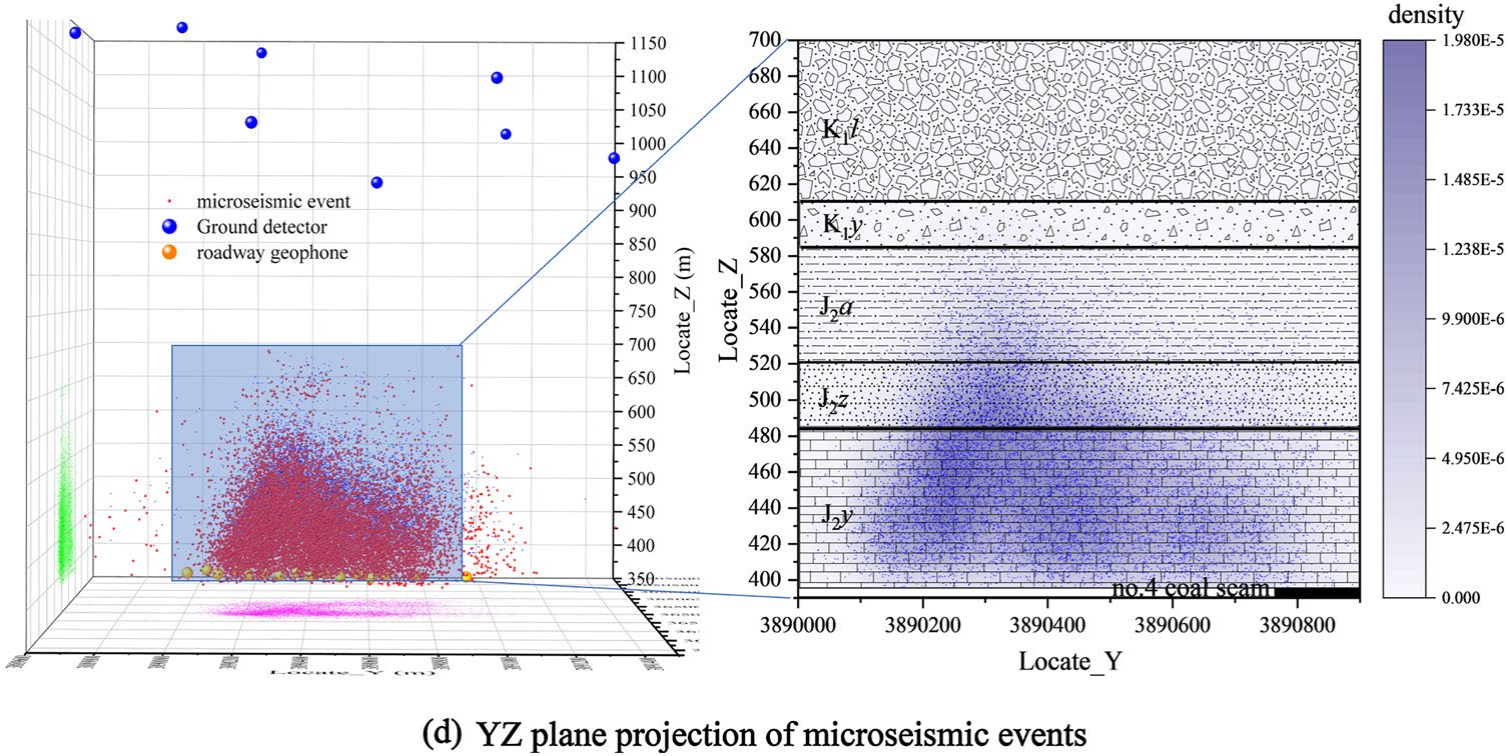
Projection map of the spatial distribution of microseismic events.

During the monitoring period, the microseismic events are projected onto the XY, XZ, and YX planes (Fig. 7, the legend indicates the elevation of microseismic events).

From Fig. 7b, c, it can be seen that the microseismic events are mainly concentrated between the 4103 working face and the 4102 working face mining hollow area, indicating that the 4102 disturbs the adjacent 4103 working face roof plate more during the process of pushing mining. Hence when the 4103 working face is mined, the roof plate rupture is mainly inclined to the 4102 mining hollow area side. A large number of microseismic events are developed, while there are also a large number of microseismic events on the 4103. At the same time, there are also a large number of microseismic events on the protected coal pillar between 4103 and 4104 working faces, but the number is significantly less than that on the side of the 4102 working face mining void area, and less on both sides directly above the working face. During the monitoring process, the elevation of the dense zone of microseismic events increased significantly after pushing the mining through the F21 fault (F21∠84°ΔH = 6.4 m).

From the analysis of the microseismic event projection maps in XZ and YZ planes, the microseismic events are mainly concentrated between elevations of 400–520 m, and the rapid decrease in development above 520 m accounts for 14.47% of the total number of microseismic events.

In the + 400~+ 450 m section, the cumulative number of microseismic events accounts for 51.8% of the total number of events, and the cumulative energy of large-energy events accounts for more than 60% of the proportion of each section, so this section is considered to be the development area of the adventitious zone. In the + 450~+ 500 m section, the cumulative energy of each section gradually decreases, and the number of microseismic events and the cumulative energy of large-energy microseismic events account for decreasing progressively, with medium-energy and small-energy microseismic events dominating. The number of microseismic events and the proportion of large-energy microseismic events decreased gradually, and the area was dominated by medium-energy and small-energy microseismic events, and the area was considered to be a rift zone development area.

**Figure 8:**
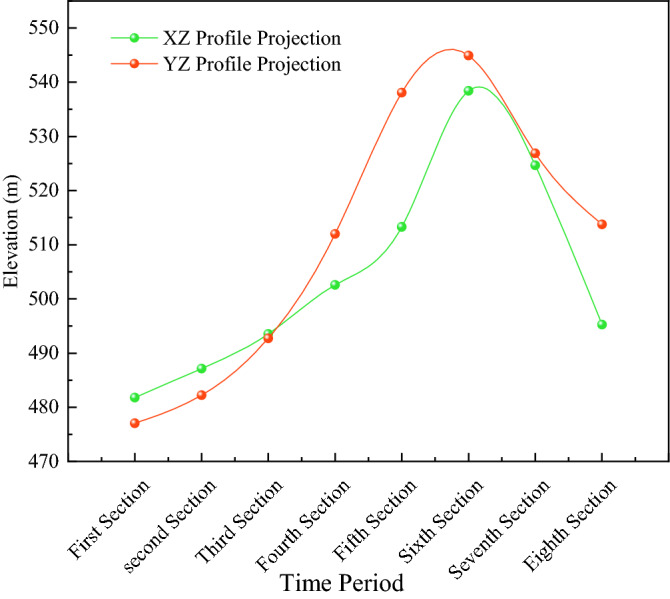
Top boundary elevation of water diversion fracture zone in different periods.

According to the development of dense zones of microseismic events in different periods (Table 2), density cloud maps on different planes (XZ and YZ planes) were drawn. Through the analysis of the cloud map, the development height of the water-conducting rift zone is directly related to the mining area formed by the pushing of the working face: when the pushing distance of the working face is smaller than the width of the working face, the development height of the water-conducting rift zone in the direction of the working face (XZ) is larger than that in the tendency (YZ); when the length of the mining area is formed by the pushing of the working face is larger than the width of the working face, the development height of the water-conducting rift zone in its tendency direction is higher. In addition, the fault zone also greatly influences the development of the water-conduction fracture zone. The rupture dense zone of the XZ face increases 35.5 m, and the rupture signal dense zone of the YZ face increases 32.91 m during the working face over the fault zone compared with the stable period, and the rupture signal dense zone returns to the stable stage when it crosses the fault for about 65 m. The water-conduction fracture zone development height of the XZ directional section is 495.27 m, and the water-conduction fracture zone development height of the YZ inclined section is 495.27 m. The development height of the hydraulic fracture zone of the XZ trending section is 495.27 m, and the development height of the hydraulic fracture zone of the YZ trending section is 512 m (Fig. 8).

**Table 2:** Similar material simulation experiment stratigraphy statistics and material ratio table.

The name and number of the rock formation		Terrane thickness (m)	Model thickness (cm)	Volume (cm^3^)	Quality(kg)	Analog material ratio		
Numbering	Terrane name					Sand	Plaster	Calcium carbonate
1	Coarse-grained sandstone	140	70	280,000	448	8	3	7
2	gravel	36	18	72,000	115.2	7	3	7
3	Argillaceous sandstone	20	10	40,000	64	7	2	8
4	mudstone	10	5	20,000	32	7	2	8
5	Coarse-grained sandstone	20	10	40,000	64	8	3	7
6	Fine-grained sandstone	20	10	40,000	64	7	3	7
7	Coarse-grained sandstone	12	6	24,000	38.4	8	3	7
8	Sandy mudstone	14	7	28,000	44.8	7	2	8
9	mudstone	22	11	44,000	70.4	7	2	8
10	Coarse-grained sandstone	14	7	28,000	44.8	8	3	7
11	Sandy Mudstone	18	9	36,000	57.6	7	2	8
12	Coarse-grained sandstone	12	6	24,000	38.4	8	3	7
13	mudstone	12	6	24,000	38.4	7	2	8
14	Fine-grained sandstone	6	3	12,000	19.2	7	3	7
15	Number 4 Coal seam	4	2	8000	12.8			
	Total	360	180	720,000	1152			

Experimental equipment height is limited, and the coal seam is buried 600 m deep. According to the model scale, the experimental equipment builds a height of 360 m, and the remaining 240 m geotechnical conversion model mass of 768 kg, with a counterweight 5.15 kg each, uses counterweight 150 blocks.

Based on the theory of mine pressure and rock control, the maximum development elevation of the water-conducting fracture zone in working face 4103 under the influence of the tectonic (no fault) layer is 512 m. The development height of the water-conducting fractured zone is about 117 m, and the fracture mining ratio is 30.79, as determined by analyzing the characteristics of the number, energy, and density of microseismic events.

## High development pattern of the water-conduction fracture zone

### Similar conditions

Similar material simulation experiments use materials with certain strength to simulate the actual rock formation, which is a simplification of the actual rock formation. Therefore t here should be a similarity in mechanical strength between the simulated rock formation and the actual rock formation.

(1) Geometric similarity ratio:

2 $$a_{l} = \frac{{x_{r} }}{{x_{m} }} = \frac{{y_{r} }}{{y_{m} }} = 200$$

where *a*_*l*_ is the geometric similarity ratio, *x*_*m*_ is the simulated rock strike length, *x*_*r*_ is the actual rock stratigraphic strike length, *y*_*r*_ is the actual cumulative thickness of the rock formation, and *y*_*m*_ is the vertical height of the simulated rock formation.

(2) Similarity ratio of capacity and weight

3 $$a_{r} = \frac{{r_{r} }}{{r_{m} }} = 1.6$$

where *a*_*r*_ is the similarity ratio of volume to weight, *r*_*r*_ is the density of the original rock, *r*_*m*_ is the similar material density.

(3) Similar ratio of stress and various strengths

4 $$a_{\sigma } = \sigma_{{\sigma_{s} }} = a_{r} \times a_{l} = 100 \times 1.6 = 160$$

where *a*_*σ*_ is the strength similarity ratio.

(4) Time similarity ratio

5 $$a_{t} = \sqrt {a_{l} } = 10$$

### Construction of similar material models

According to the general characteristics of the stratigraphic structure in the coal-endowed area, river sand is used as aggregate, gypsum as cement and large white powder as filler, and different ratios are used to simulate the soft, medium-hard and hard rock layers in the stratum^13,22^. White mica flakes were used to simulate the laminated surfaces between each rock layer. Based on the similarity ratio and the physical and mechanical parameters of the simulated coal seams (mainly compressive strength and elastic modulus, supplemented by other parameters), the formulations and ratios of similar materials were selected in combination with the test results of similar material specimens^23,24^.

Due to the different thickness, capacity and proportioning formula of each simulated rock layer, the total weight of materials used for laying each simulated rock layer and the amount of various materials vary. In this experiment, the total weight of each layered material was calculated according to the following formula, and then the amount of various materials was calculated according to the proportioning formula.

6 $$W = k \times l \times d \times h \times \gamma_{m}$$

where *W* is the total weight of the material; *k* is the material loss factor: *l*, *d*, *h* are the length, width and thickness of the layer respectively; *γ*_*m*_ is the density of the layer of similar materials.

According to the objective of a similar material simulation experiment, it was determined that the data 7-5 drilling holes was the main one to the stratum of this similar material simulation, based on the complete analysis of geological data from the first mining area and drilling data within the range of 4103 working face Table 2.

**Figure 9:**
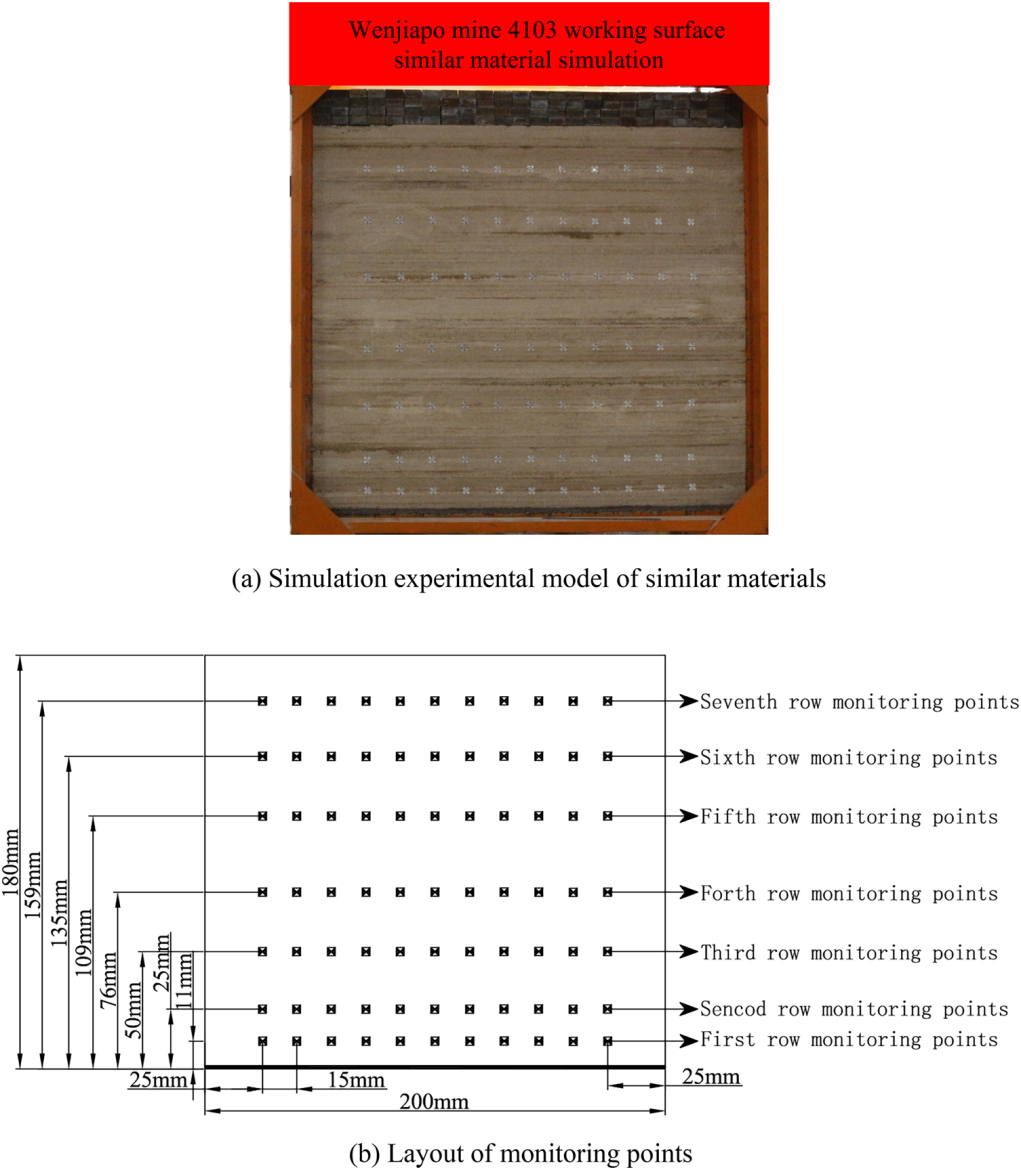
Similar material simulation experiment and monitoring scheme.

The device used for a similar material simulation experiment is 2.0 m long, 1.8 m high, and 0.2 m thick, simulating the stratum. Combined with similar conditions, it was determined that this experiment simulates the development process of three zones on the coal seam strike profile with a ratio of 1:200. Due to the limitation of the simulation experiment device, the full stratum structure was not simulated, and the stratum 90 m below the surface was realized by external loading (Fig. 9a; Table 3). To match the actual mining, the left and right boundaries of the model were left with 20 cm coal pillars each, and the length of the mining area was 160 cm, which was excavated in 16 steps with 10 cm per step.

**Table 3:** Model size and mining conditions.

Category	Lateral length	Vertical height	Overburden thickness	Coal pillar width	Coal seam mining height
Prototype size/m	400	360	356	40	4
Model size/cm	200	180	178	20	2

To obtain high precision displacement measurement values, a total station and vic-3D imaging technology were used in the simulation experiment to monitor the displacement and strain of the model.

From the top of the coal seam to the seventh-row, there are seven rows of monitoring points in the overburden ,and the distance between each row is 15 cm. The first row is 11 cm from the bottom of the coal seam, the second row is 25 cm from the bottom of the coal seam, the third row is 50 cm from the bottom of the coal seam, the fourth row is 76 cm from the bottom of the coal seam,and the fifth row is 109 cm from the bottom of the coal seam, the sixth row is 135 cm from the bottom of the coal seam, the seventh row is 159 cm from the bottom of the coal seam. The seventh row is 159 cm away from the coal seam floor, this is conducted mainly for monitoring and recording the overburden subsidence value after excavation of the coal seam (Fig. 9b).

### Roof overburden fracture development pattern

The working face of 4103 is 245 m wide, leaving about a 40 m section of coal pillars. After the working face was mined, the fissure development pattern derived from the experimental photo processing is shown in Fig. 11. After the working face is fully mined, the total length of the collapsed body is 133 cm, the height of the overlying rock upward fissure is 75 cm, the height of the caving zone is 15 cm, the height of the fracture zone is 42.8 cm, and the height of the water-conducting fracture zone is 57.8 cm, which is 115.60 m. It reaches the bottom of the water barrier of Jurassic Middle Formation Anding Formation, which is 30.47 times the mining height. The collapse angle of rock on the open-cut side and the working face side are both 36°, and the fissure angle is 54°.

**Figure 10:**
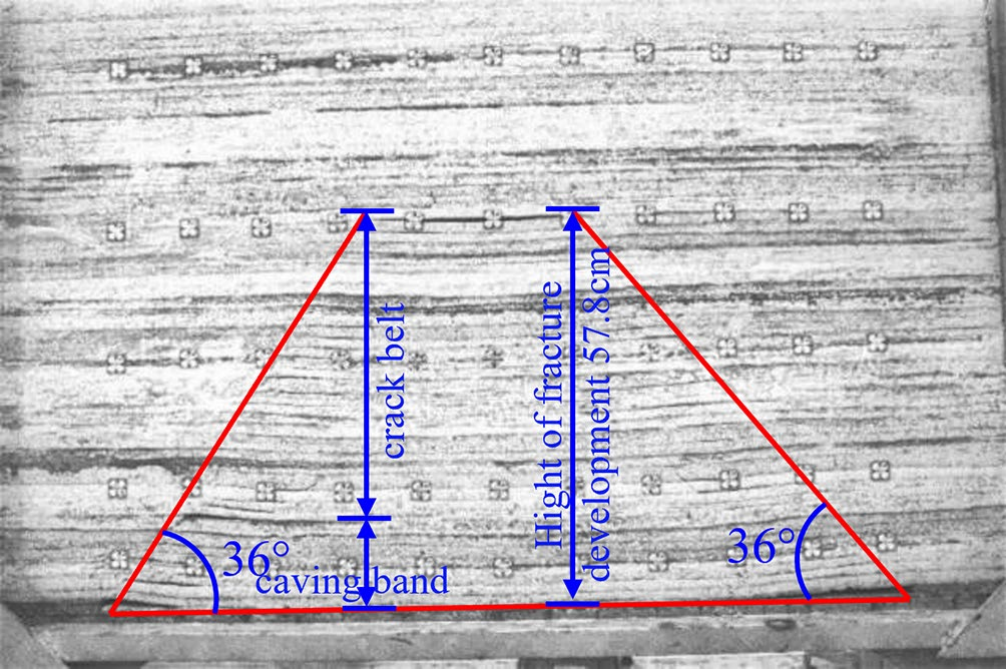
Caving diagram of advancing 160 cm overburden in the working face.

From the analysis of the density map of microseismic events in the strike direction of the working face (Fig. 10), the area with a higher density of microseismic events at the front of the working face forms an angle of 36° with the coal seam floor, which is consistent with the results of the similar material simulation.

### Overlying rock lead deformation analysis

**Figure 11:**
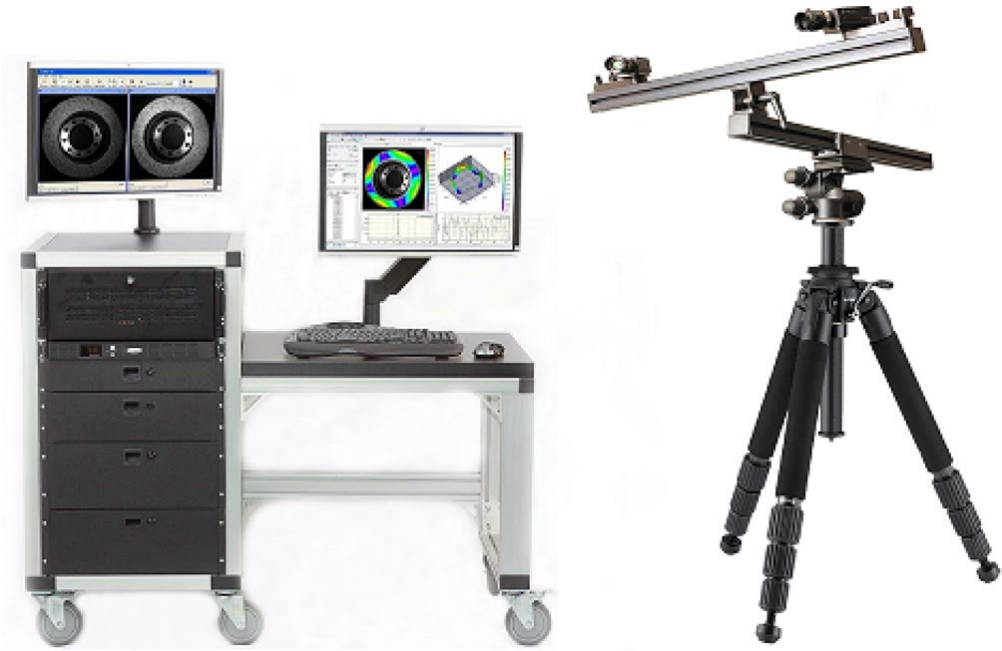
The vic-3D imaging equipment.

In this experiment, deformation was monitored using two methods: total station monitoring and vic-3D imaging (Fig. 11) technology monitoring. Five rows of monitoring points were arranged on the model’s surface for total station monitoring, among which the fifth row of monitoring points was distributed on the upper boundary of the model, mainly for monitoring the surface deformation, and the distance between each point was 10 cm.

Vic-3D imaging technology monitoring calibrates the model by applying scatter spots on the surface of the model. The model is continuously photographed during the model development process, and the monitoring system superimposes and calculates the adjacent pictures to draw the model deformation cloud map and contour map to monitor the model deformation.

**Figure 12:**
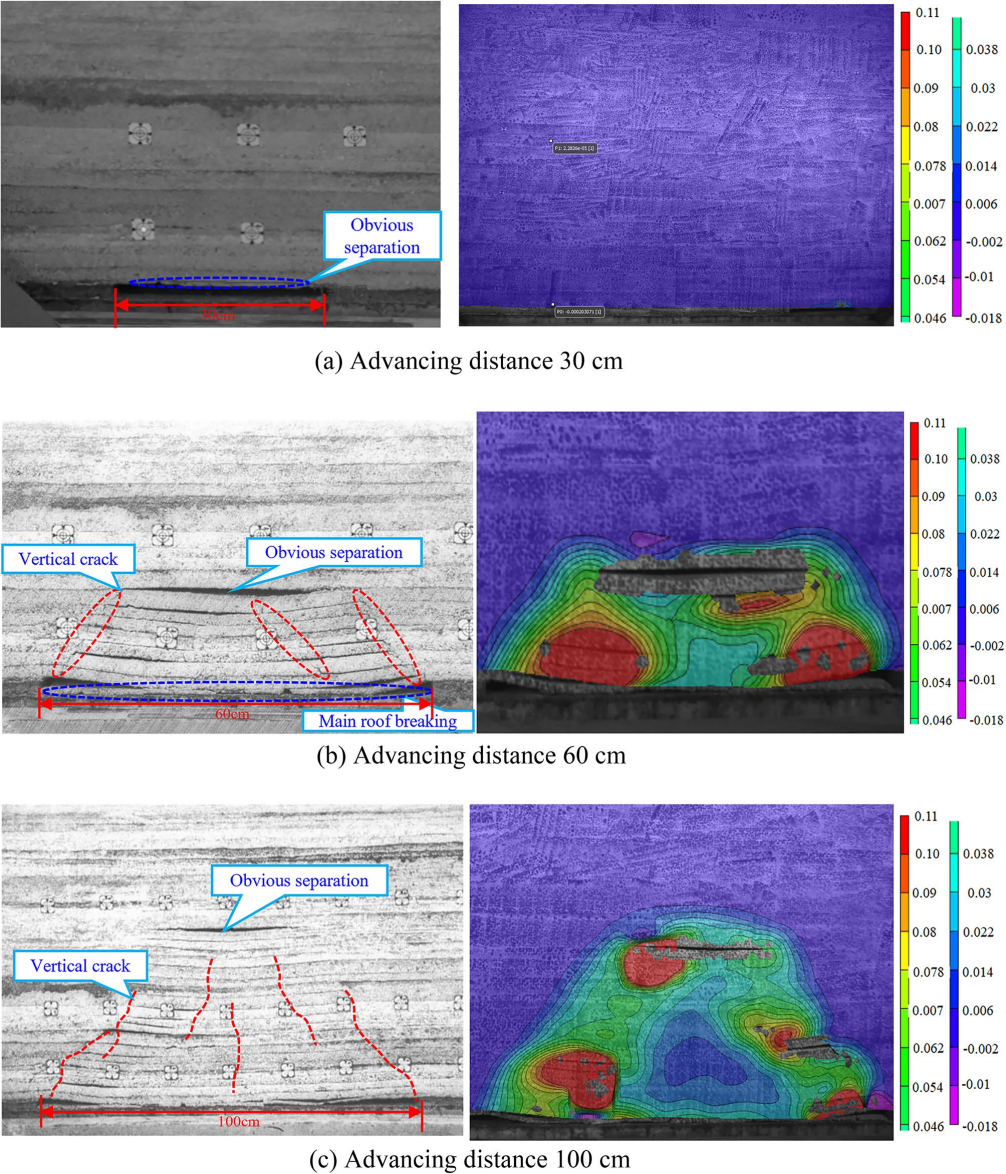

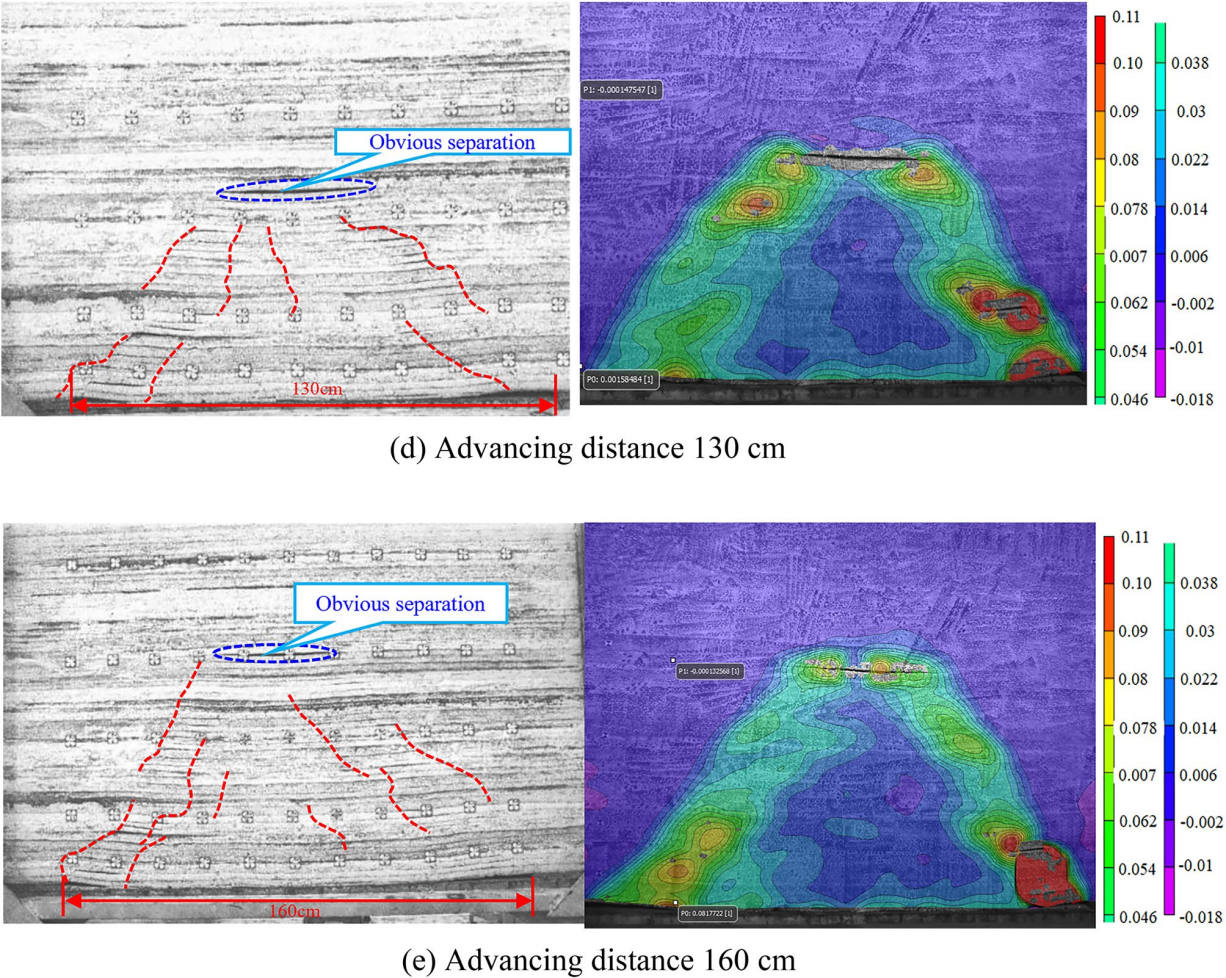
Vertical deformation and fractures evolution of overlying strata.

The vic-3D imaging technology was used to monitor the overburden strain after mining the simulated coal seam. Selected major steps to analyze the vertical strain and variation of the overlying bedrock. The strain is the relative deformation value of the material under the action of the external force. The lead direction strain represents the vertical elongation or compression rate, which is a dimensionless number without a unit. Positive values represent elongation of the object under external forces, while negative values represent compression (Fig. 12).

When mining at a length of 30 cm, 8 cm from the cut and 3 cm from the coal seam floor, the top plate of the coal seam develops an 18 cm long abscission layer. Because the abscission layer space is too small, there is no response in the strain cloud diagram (Fig. 12a).

Advancing distance 60 cm, the coal seam roof is pressed for the first time, and the weighting step distance is 27 cm. The coal seam top plate collapses from the separate layer, collapses 27 cm in length and 3 cm in thickness, 27 cm from the cut, and comes into contact with the coal seam floor to form a 23 cm long cantilever segment. The overburden deformation cloud map shows that the left side is near the fracture zone at the working face, the right side is near the fracture zone at the cut, the lower part is the bottom of the coal seam that has been mined, and the upper part is near the maximum height of the abscission layer space. The strain cloud map pattern is basically consistent with the overburden collapse pattern. At this time, the height of the fall band is 15 cm, the height of the fracture zone is 1.6 cm, and the height of the water-conducting fracture zone is 16.6 cm (Fig. 12b).

The accumulated mining length is 100 cm, part of the fissure closure disappeared, the third of incoming periodic pressure, the pressure step is 22.5 cm, the maximum collapse height is 43.8 cm, and the total collapse length is 73.5 cm. A distance of 72.5 cm from the cut forms a cantilevered segment 21.5 cm long. The overlying bedrock deformation cloud map shows that the left side is near the water-conduction fracture zone at the working face, and the right side is near the water-conducting fracture zone at the cut. The lower part is the bottom plate of the coal seam that has been mined, and the upper part is near the maximum height of the deviated seam development. Its strain cloud map pattern is basically consistent with the overlying rock collapse pattern.At this point, the falling band height is 15 cm, the crack band height is 28.8 cm, and the water-conduction fracture zone height is 43.8 cm (Fig. 12c).

When the cumulative mining length is 130 cm, a new abscission layer space is developed and part of the fracture closes and disappears, with a total body collapse length of 103.7 cm, a maximum collapse height of 53.4 cm and an inclination of 13°. The left side of the overburden strain cloud is near the fracture zone at the working face, and the right side is near the fracture zone at the cut. The lower part is the bottom plate of the mined coal seam, and the upper part is near the location of off-bed development. The strain cloud pattern is basically consistent with the overburden collapse pattern. At this time, the height of the caving band is 15 cm, the height of the fracture zone is 38.4 cm, and the height of the water-conduction fracture zone is 53.4 cm (Fig. 12d).

When the cumulative propulsion length is 160 cm, the previously formed abscission layer space has collapsed. However, several new abscission layer spaces have been formed, a split gap has been closed, and the morphology of the bedrock strain cloud map is consistent with that of the strain cloud map at mining to 130 cm. Finally, the upper bedrock stress cloud map shows that the falling zone height is 15 cm, the crack band height is 42.8 cm and the development height of the water-conduction fracture zone is 57.8 cm, all of which are consistent with the model cover rock collapse pattern. There was only one tension deformation in the local area near the cutting eye, with the maximum tension strain in the lead direction being 0.11 and the maximum compression strain being 0.018, indicating that the maximum tension amplitude was greater than the maximum compression amplitude and that the transition from the compression area to the tension area was gradual. It is a slow and steady process (Fig. 12e).

### Overlying rock subsidence analysis

**Figure 13:**
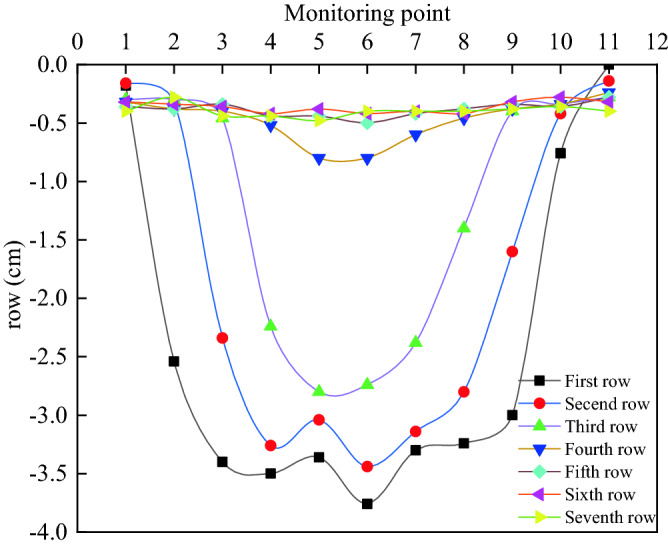
Subsidence and displacement map for each row of measuring points after the end of the excavation.

By plotting the converted final subsidence change curve for each row of measurement points (Fig. 13), it is more obvious to see the overburden collapse pattern and the collapse range of each row of measurement points after the end of mining. The closer to the working face, the greater the overburden sinking value, the first to the third row apparently collapse, the fourth row deformation is more apparent, and in the bending deformation more apparent stage, the fifth to the seventh row deformation is not evident. The sinking curve is roughly symmetrical with measurement point No. 6, and the maximum sinking value of measurement point No. 6 in the first row is 1.88 cm, which is 3.76 m.

### Analysis of the height development pattern of the water-conducting fracture zone

**Figure 14:**
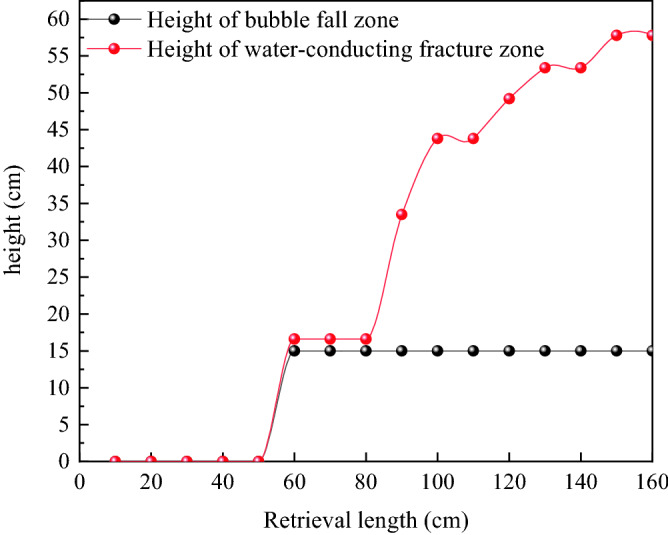
Relationship between propulsion distance and height of caving zone, fracture zone and water diversion fracture zone.

Through continuous observation and recording of displacement changes in the observation points during the advancing process of the working face, the relationship table was compiled to analyze the advancing distance of the working face and the height of the height of the caving zone, the height of the fracture zone, and the height of the water-conduction fracture zone. In addition, the curve diagram was drawn to analyze the advancing distance of the working face and the height of the caving zone, the height of the height of the caving zone and the height of the water-conduction fracture zone (Fig. 14).

The water-conducting fracture zone is composed of the height of the caving zone and fracture zone. As the working face advances, the overlying rock damage height is basically a horizontal straight line, the overlying rock damage degree is not obvious, and has been in the bending deformation stage. When the working face advances to 60 cm, the overburden comes to pressure for the first time and the height of the collapse body is 4.8 cm. When the working face advances to 70 m, the damage height of the overburden rock layer appears rapid growth and water-conduction fracture zone. The height of the riser belt reaches 15 cm, with the coal seam mining, the height of the riser belt no longer increases; when the advancing distance reaches 100 cm, the growth rate of damage height slows down. In addition, 57.8 cm is the maximum water-conducting fracture zone height at an advance of the work surface to 160 cm. The fissure ratio reaches a maximum of 30.42 when the working face is advanced to 160 cm.

On the whole, it seems that as the working face advancement distance increases, the development height of the water-conducting fracture zone presents a step-like curve. This is because it is primarily mainly affected by the periodic destruction and deformation of the overlying rock layer.

## Conclusion

1. Compared with the previous method of detecting the water-conducting fracture zone, a microseismic monitoring system can better respond to the spatial pattern and development process of fissure development in the process of coal mining and provide a scientific basis for fine water control work.
2. The microseismic monitoring results show that the roof fracture is mainly distributed on the side of the working face adjacent to the mining area, followed by the side of the adjacent successive working faces, while the fissures directly above the working face have relatively less spatial distribution characteristics.
3. The development process of the hydraulic fracture zone was defined by the dense zone of microseismic events in different periods using the joint " the underground—ground " microseismic monitoring data. The results show that the maximum elevation of water-conducting fracture zone development is 512 m without tectonic influence, which is 35.5 m lower than that of fault influence, and the fracture -to-mining ratio is 30.79 times when the mining height is 3.8 m.
4. The maximum height of water-conducting fracture zone development at the working face obtained from similar materials is 57 cm. comparing this conclusion with the analysis results of microseismic monitoring data, the maximum value of water-conducting fracture zone development derived from both is basically the same.

## Supplementary Information

Supplementary Information 1.

Supplementary Information 2.

## Data Availability

All data generated or analysed during this study are included in this article and its supplementary information files.
